# RootBot: High‐throughput root stress phenotyping robot

**DOI:** 10.1002/aps3.11541

**Published:** 2023-08-28

**Authors:** Mia Ruppel, Sven K. Nelson, Grace Sidberry, Madison Mitchell, Daniel Kick, Shawn K. Thomas, Katherine E. Guill, Melvin J. Oliver, Jacob D. Washburn

**Affiliations:** ^1^ Department of Biomedical, Biological, and Chemical Engineering University of Missouri Columbia Missouri USA; ^2^ Director of Plant Science Heliponix, LLC Evansville Indiana USA; ^3^ Plant Genetics Research Unit USDA‐ARS Columbia Missouri USA; ^4^ Division of Plant Science and Technology University of Missouri Columbia Missouri USA; ^5^ Division of Biological Sciences University of Missouri Columbia Missouri USA

**Keywords:** automation, drought stress, phenotyping, roots

## Abstract

**Premise:**

Higher temperatures across the globe are causing an increase in the frequency and severity of droughts. In agricultural crops, this results in reduced yields, financial losses, and increased food costs at the supermarket. Root growth maintenance in drying soils plays a major role in a plant's ability to survive and perform under drought, but phenotyping root growth is extremely difficult due to roots being under the soil.

**Methods and Results:**

RootBot is an automated high‐throughput phenotyping robot that eliminates many of the difficulties and reduces the time required for performing drought‐stress studies on primary roots. RootBot simulates root growth conditions using transparent plates to create a gap that is filled with soil and polyethylene glycol (PEG) to simulate low soil moisture. RootBot has a gantry system with vertical slots to hold the transparent plates, which theoretically allows for evaluating more than 50 plates at a time. Software pipelines were also co‐opted, developed, tested, and extensively refined for running the RootBot imaging process, storing and organizing the images, and analyzing and extracting data.

**Conclusions:**

The RootBot platform and the lessons learned from its design and testing represent a valuable resource for better understanding drought tolerance mechanisms in roots, as well as for identifying breeding and genetic engineering targets for crop plants.

In the past half‐century, there has been a substantial increase in drought and extreme temperature events across the United States, resulting in significant impacts on agricultural yield and threatening food production and security across the globe (Mazdiyasni and AghaKouchak, 2015; Zipper et al., 2016). In the past three decades, maize (*Zea mays* L.) suffered an average yield reduction of 39% in scenarios of 40% water reduction (Daryanto et al., 2016). In the United States, the production of maize is a multi‐billion‐dollar industry, with the United States leading in both the production and consumption of maize worldwide (Erenstein et al., 2022). The economic importance of maize and its high susceptibility to yield loss during drought underscore the importance of better understanding the genetic and physiological mechanisms that could be used to improve drought tolerance and develop interventions against drought‐induced crop loss.

Plant roots are primarily responsible for the uptake of water and nutrients from the surrounding soil, and are therefore critical to understanding and combating the effects of drought stress. When roots are exposed to a water deficit, their growth regulation is generally considered to be dependent upon three main factors: (1) abscisic acid accumulation, (2) cell wall extension, and (3) osmotic modification (Yamaguchi and Sharp, 2010). Maize typically responds to drought stress by temporarily inhibiting the growth of the shoot while simultaneously maintaining the growth of roots, a survival mechanism that allows the roots to grow deeper and potentially find more water (Li et al., 2015). Although this is not sustainable for the plant in the long term, short‐term maintenance of root growth may be an important drought‐tolerance mechanism for breeding into elite cultivars (McGrail et al., 2020; Tracy et al., 2020).

Many drought‐stress studies in maize have exclusively focused on the shoot and aboveground components due to their ease of analysis, rather than examining underground plant components (Maeght et al., 2013; Li et al., 2015). Other studies have investigated roots and their responses to the environment using simplified systems such as transparent containers and liquid substrates (e.g., agar or hydroponics) to easily visualize and quantify root growth (Le Marié et al., 2014; Judd et al., 2015). However, liquid media or solid agar are not ideal environments for roots and do not necessarily mimic what is observed when roots are grown in soil. Observations have shown that morphological changes in roots can result from differences in solute diffusion, gas exchange properties, and exposure to light while using media like agar or hydroponic solutions (Rich and Watt, 2013; Robbins and Dinneny, 2015; Nelson and Oliver, 2017). This can lead to confounding effects when analyzing roots under drought stress.

A major challenge with studying roots, both in the field and under controlled conditions, is the complexity of the root system and the challenges of measuring it. Significant progress has been made in the development of systems for imaging roots and systems for measuring the roots in those images or three‐dimensional (3D) reconstructions (Wasaya et al., 2018). For example, several well‐validated and continuously developed software packages exist for measuring roots from different types of images and image reconstructions (Lobet et al., 2011; Galkovskyi et al., 2012; Das et al., 2015; Nelson and Oliver, 2017; Liu et al., 2021). Additionally, systems such as minirhizotrons have enabled some advancements in field‐based root phenotyping, although most field‐based root phenotyping is still done via “shovelomics” (Trachsel et al., 2010; Gray et al., 2013; Yu et al., 2020; Hui et al., 2022). The excavation of roots in the field, followed by imaging in the lab using standard cameras, X‐ray machines, or other more sophisticated multi‐camera systems, remains the state of the art for field‐based root trait measurement (Gerth et al., 2021; Liu et al., 2021). Non‐invasive image‐based root phenotyping methods have also been reported but may not represent direct measures of root growth (Peruzzo et al., 2020). Although many advances have been made in image‐based root phenotyping, no single method is ideal for every situation.

Another important consideration when developing a methodology to study roots is ensuring the root is not exposed to white light, as this can activate photoreceptors that impact root growth (Lee et al., 2016). Keeping roots in continuous darkness and only exposing them to dim green light for phenotyping and imaging can prevent any spurious impacts on root light responses (Nelson and Oliver, 2017). A final major consideration when developing a drought‐stress study is the need to quantify the water deficit experienced by roots in order to accurately interpret the data obtained. The most accepted method for achieving a quantifiable water deficit is by measuring the water potential ($\psi_{w}$) of the substrate (Robbins and Dinneny, 2015; Lee et al., 2016). Nelson and Oliver (2017) developed a soil‐based plate method for analyzing roots under drought stress. The main advantages of this method are the use of actual soil or potting media as opposed to agar, the ability to contain that soil and the growing roots in a closed plate system while maintaining humidity, and the use of polyethylene glycol (PEG) to induce water deficit stress in a predictable and quantifiable manner (Nelson and Oliver, 2017). Using a system of boxes, a dark room, green lights, and a digital camera, they demonstrated the utility of this system for the quantification of roots under well‐watered and water‐stressed conditions. They also showed that this plate system could be used for roots from a number of species including wheat, maize, and soybean (Nelson and Oliver, 2017). However, this method was extremely time intensive and limited by labor costs in the number of times roots could be imaged.

In this study, we address the current gap in research methods and systems for studying root responses to water stress in a high‐throughput manner using controlled conditions that closely replicate field environments. We streamlined the method from Nelson and Oliver (2017) by automating the plate handling, photography, and analysis using a custom‐built system coined “RootBot” (Figure 1). RootBot stores self‐contained soil plates in vertical slots for root growth and uses a gantry system to move each plate to a camera for automatic photographing at pre‐programmed time intervals. RootBot is housed inside a growth chamber, which allows the temperature and lighting to be carefully controlled. The RootBot method is particularly advantageous for time‐course experiments, where multiple images are captured in a series to evaluate the rate of growth and root length at various time points. Another advantage of this system is its potential to house over 50 plates at one time, enabling the study of root responses to drought and/or temperature stress on a high‐throughput scale with significantly reduced labor costs. By utilizing high‐throughput phenotyping, we ultimately intend to improve understanding of the genetic factors involved in maize growth to better target desirable traits for this and other crops (Mir et al., 2019; Peruzzo et al., 2020). While we focus on maize in this study, the system could theoretically be used with any plant species that can be germinated from seeds.

**Figure 1:**
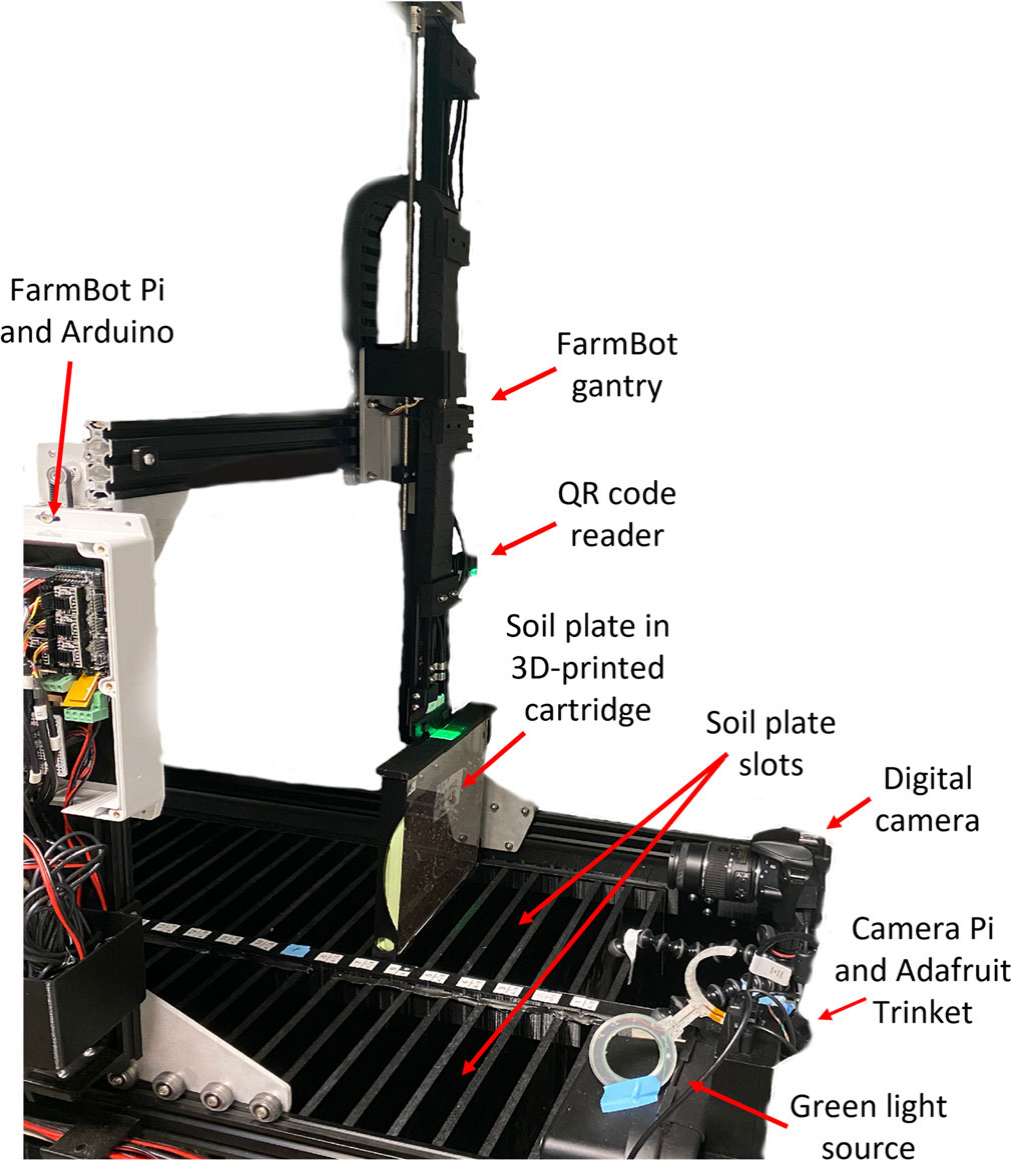
The RootBot phenotyping system as seen from the side. Pi = Raspberry Pi.

## METHODS AND RESULTS

### Overall RootBot workflow

**Figure 2:**
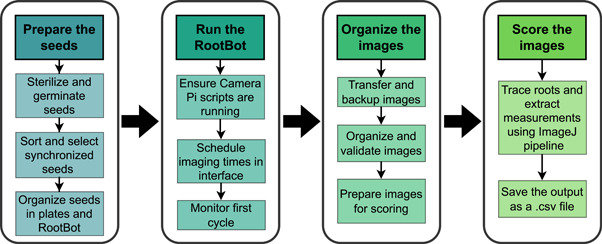
RootBot workflow diagram. The RootBot system works in four steps: (1) preparing and organizing the seeds for use in the RootBot, (2) placing the seeds in the RootBot and running the phenotyping experiment, (3) organizing the images generated by the RootBot, and (4) scoring the images from the RootBot to extract quantitative root measurements.

The complete process of root phenotyping in the RootBot system involves multiple seed preparation, root imaging, and analysis steps (Figure 2). First, the desired seeds are prepared following the methods described by Nelson and Oliver (2017). These methods, as well as the use of different soils, water deficit conditions, or other factors, can be modified by the user in many ways depending on the desired experiment. Once the seeds are properly prepared, organized, and placed within their plates with the embryo side facing the transparent imaging surface, the plates are placed within specially designed cassettes (described below) and inserted into RootBot's robotic gantry system. The RootBot system images the plates based on the experimental design designated by the user, and the images are organized and analyzed. The design and use of the RootBot system and image analysis pipeline are described in detail below.

To test and improve the RootBot system, diverse genotypes from the maize 282 association panel (Flint‐Garcia et al., 2005) were germinated, imaged, and phenotyped for primary root length as described below. In order to load the RootBot with seeds that are at similar stages of germination and growth, approximately 25 seeds were germinated for each desired plate. After germination, the 10 seeds with the most similar root lengths and size were selected for use and the remaining 15 were discarded (see Nelson and Oliver, 2017 for more details).

### RootBot mechanical engineering and hardware design

The RootBot was heavily inspired by FarmBot, the popular automated home gardening system (https://farm.bot/). The open design for the FarmBot Genesis v1.4 system was used as a starting point for the RootBot design and was modified to replace the normal soil bed with an acrylic base containing two rows of 38 slots designed to hold transparent soil plants in a vertical position (Appendices 1 and S1). Each soil plate was 240 mm × 240 mm × 1.7 mm (item #240845; Thermo Fisher Scientific, Waltham, Massachusetts, USA). As the FarmBot designs, 3D models, and software are all open source, various FarmBot components were customized to fit our needs. All of the 3D designs for this project can be found in the BitBucket repository (see Data Availability Statement). The FarmBot operating system was installed on a dedicated Raspberry Pi (https://www.raspberrypi.org; Raspberry Pi Foundation, Cambridge, United Kingdom) positioned on the gantry, while a second Raspberry Pi was used to capture the image via custom Python scripts (Camera Pi). An audio cable was utilized as the communication line between the two Raspberry Pis. When the Camera Pi received this audio signal, the custom Python scripts were activated, and an image was captured. The FarmBot gantry system was modified with 3D‐printed parts including a two‐pronged hook system to grasp and lift the soil plates from the slots (Figure 1). Cartridges were designed and 3D printed to hold the soil plates and enable an easy connection and support for lifting them with the gantry system (Appendix S2). A digital camera (Nikon D3300 DSLR camera with 18–55‐mm lens; Nikon, Tokyo, Japan) was mounted at a fixed location on one end of the system, and a green illumination light (NeoPixel Ring, 24 × 5050 RGB LED, customized to include only green LEDs; Adafruit, New York, New York, USA) was mounted near the camera to illuminate the plates for imaging without influencing root growth (Nelson and Oliver, 2017). A small microcontroller (Adafruit Trinket 5 V with CircuitPython; Adafruit) was used to control the NeoPixel LED ring. The microcontroller was triggered by a general‐purpose input/output (GPIO) signal sent by the Camera Pi to turn the LED on and off. LEDs have a narrow range of light wavelengths and, in this case, were set up to activate momentarily for imaging at a very dim level. An additional small camera was mounted on the gantry oriented downwards in the direction of the plates to read QR code adhesive labels placed on the top of each plate and enable identification of the plates via custom‐designed software (described below). The QR code unique ID for a given capture is automatically integrated into the filename of the output image to allow easy identification. The RootBot was designed to pick up the plates one at a time, move them to the camera for imaging, and return them to their slots. The entire system was designed to fit precisely within a small growth chamber to control the temperature and prevent exposure to external light. Because each plate used for root growth measurements was sealed, the chamber's humidity had no direct impact on root growth conditions and was not controlled by the chamber.

### Software design, co‐option, and integration

The RootBot was designed to co‐opt and take advantage of the FarmBot system's well‐designed gantry control system and software while also utilizing additional scripts and custom software on an external Raspberry Pi (distinguished as the Camera Pi hereafter) to interact with the FarmBot system and control the camera, green light source, image file naming, and related imaging tasks. Custom commands and plate location coordinates (using the *x*, *y*, *z* Cartesian coordinate system) were programmed into the open‐source FarmBot OS software to schedule the pickup, movement, triggering of the light and camera (through an audio signal sent to the Camera Pi), and return of plates to their designated slots after imaging (detailed instructions are available on protocols.io [Ruppel et al., 2023a]; see the Data Availability Statement). The Camera Pi was designed and programmed to detect a signal from the FarmBot system. Upon receipt of the signal, Camera Pi (1) sent a signal to the Adafruit Trinket microcontroller controlling the light source to activate the green light, (2) captured the QR code on the top of the plate using the QR code camera, (3) decoded the plate number from the QR code to ascertain which plate is being imaged, (4) captured an image of the plate's roots and soil contents through the transparent plate, and (5) saved the image using the information from the QR code as well as the time and date (see code repository). Through extensive testing of the system, many refinements were made to the customized FarmBot commands, image sequencing, and storage system to increase the automation and dependability of the system. One such refinement involved the addition of a QR code label to the upper right‐hand corner of the imaged surface of the plate so that the plate number and information could be verified directly from the same image used for root scoring in the subsequent analysis process. In future versions of the RootBot, the camera for reading the QR code label on the top of the plate could be eliminated as this refinement is redundant.

### Data analysis pipeline

Custom scripts were designed to organize, store, and analyze images from the RootBot, and these images were transferred to a separate computer for storage using *rsync*, a popular Linux command‐line tool. A custom Python script, *RootBotCameraPiPhotoScript.py*, was designed to sort photographs by experimental batch, plate number, and collection time relative to the start of the experiment. Key information (e.g., file creation time, plate number) is stored in each image's filename. The sorting script used the creation time of each file to sort images in an “inbox” folder into experimental batches assuming files created within a span of 48 h belonged to the same experimental batch. Within each batch, files with a difference in creation time above 2 h were assumed to belong to different time points. The oldest creation time within a batch was used to calculate the time points for all subsequent measurements. Folders were created for each time point and plate within a batch, and unrecognized plates (e.g., due to an occluded QR code) were grouped together into a separate folder for manual review. This structure organized observations but did not allow for rapid selection and copying of images (i.e., required navigating subfolders to select the desired time points). To avoid this, the script also generated a text file with the file paths of all new images taken at the desired time points (we used 6 and 36 h) to be used for transferring image batches with *rsync* if further analysis was desired. Images for the desired time points were imported into a custom‐designed ImageJ/Fiji (Schindelin et al., 2012) pipeline for processing.

**Figure 3:**
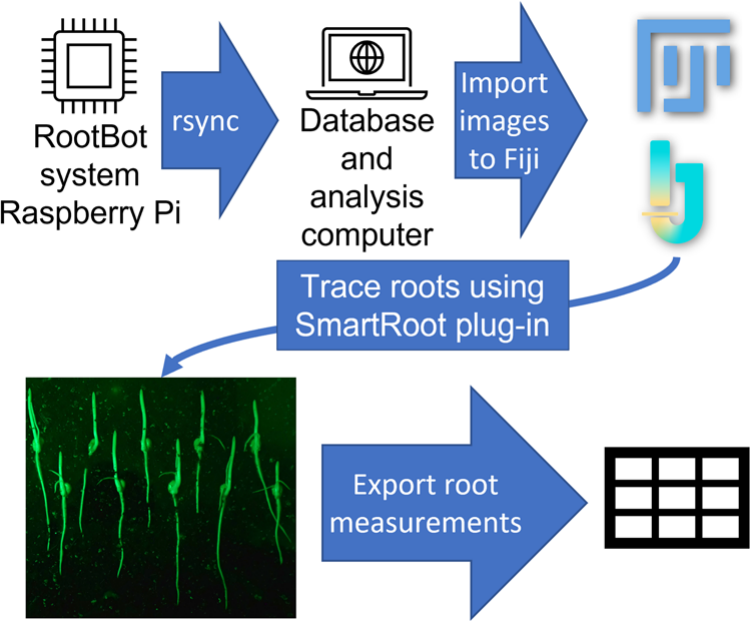
Workflow diagram illustrating the basic procedure for extracting root measurements from RootBot images.

**Figure 4:**
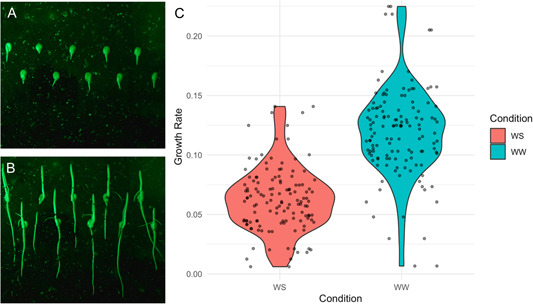
Example plate images taken by the RootBot at 6 h (A) and 36 h (B) post‐germination under well‐watered conditions. (C) Chart showing the growth rate as extracted from RootBot images of several maize inbred lines between the 6‐h and 36‐h post‐germination interval under well‐watered (WW) and water‐stressed (WS) conditions.

Image processing was accomplished via a Fiji plug‐in known as SmartRoot (Lobet et al., 2011). A detailed step‐by‐step explanation and protocol for the process are available on protocols.io (Ruppel et al., 2023b; see the Data Availability Statement). With the SmartRoot plug‐in, images were adjusted for resolution and image settings and corrected for the size of objects corresponding to measurement units in Fiji to ensure each image was compared in as uniform a manner as possible. Images were then verified for label details in the image and ID, and roots were labeled and traced (Figure 3, Appendix S3). This information was exported to a .csv file for further quantitative and statistical analysis. This spreadsheet‐like file stores the name and length (in centimeters) of each root traced. Figure 4 shows example images taken by RootBot and a summary of data extracted from multiple processed RootBot images. Generally, most of the roots in a plate could be scored; however, occasionally a root grew to the side rather than down, became entangled with another root next to it, or grew away from the transparent surface of the plate so that it could not be confidently scored and had to be discarded from the data.

### Advantages and disadvantages of RootBot

The overall cost of the RootBot is estimated at $3500 USD depending on the exact setup desired. The major drivers of cost include the acrylic base (~$1200), the main camera setup (~$500), and the FarmBot motor kit (~$400). The remainder of the cost includes a plethora of nuts, screws, belts, wheels, tracks, bearings, wires, and adapters (~$1000 for all), as well as the LED ring light (~$20), 12 V power supply (~$50), and two Raspberry Pi 3 Model Bs (~$80). All of the custom parts are 3D printed, and this can be accomplished by purchasing a printer or simply paying a service to print the parts. We estimate this cost at between $300 and $700 depending on the service used, the number of cartridges printed, and the filament utilized. CAD designs, specifications for the acrylic base, and all other details needed to construct and operate the RootBot can be found in Appendix S1 and the code repository (https://bitbucket.org/washjake/rootbot/src/master/; see Data Availability Statement). An additional consideration is the need for an enclosure to prevent root exposure to external light. To accomplish this, we built the RootBot inside a small growth chamber. The design of the base could easily be altered to fit into any standard growth chamber, and the gantry dimensions can also be adjusted to meet any size constraints.

The main disadvantage of the system, and many other growth chamber–based methods, is its limitation to the first 72 h of growth. This 72‐h time limit is based on maize. Other species, such as wheat, do not grow as quickly as maize and therefore could be analyzed over a longer time frame. Although this system was primarily designed to focus on time course traits such as primary root growth, other traits such as root thickness, root depth, or the development of lateral and seminal roots could be studied with slight alterations.

Another notable disadvantage of this system is the fact that it has more mechanical moving parts than root imaging systems where the soil plates are stationary and the camera is the only moving part, and thus presents more opportunities for mechanical failure. However, the principal advantage of the RootBot system is the ability to image many more plates (perhaps 5–10 times as many, depending on the system) within a smaller footprint and/or time frame than systems with stationary plates and a moving camera. With this larger capacity, the primary limiting factor is shifted from the number of plates one can photograph within a given time, to the labor required to prepare the plates for insertion into the RootBot system. With this semi‐automated method, less labor is required for the photography and organizational steps, however. As an example from our experience, it takes approximately 5 h of labor for a team of two people to prepare and load 12 plates into the RootBot. This includes the time required to prepare the seeds for germination, sort the germinated seeds, mix the solutions and soil, and place the seeds in the plates.

Another advantage of the RootBot (and other plate‐based root measurement systems) is its use of images and software for root measurements rather than rulers, measuring tapes, calipers, or other handheld measuring devices. The use of images and software for measurement has several clear and well‐demonstrated advantages (Lobet et al., 2011; Das et al., 2015; Nelson and Oliver, 2017; Liu et al., 2021). First, images are preserved after the roots are discarded, allowing researchers to return to the image at any point for re‐scoring by a different individual or software, or to determine the validity of any data point. Second, the measurements are placed directly in the data file by the software program, removing the error‐prone tasks of reading measurements from a measuring device and writing them down or typing them into a computer. Third, the software can accurately measure the turns and contours of the roots (similar to taking a string, lining it up with the root, cutting it to the same length as the root, and stretching it out next to a ruler to get the final length). This allows for higher accuracy measurements than would be possible with any handheld measuring device. The only time these accuracies might be poorer than a handheld device is in a fully automated scoring system where the software selects the roots without oversight from a human operator. The pros and cons of this type of system have been discussed elsewhere (Nelson and Oliver, 2017). Here, a semi‐automated system was used ensuring that each root was selected and validated by a human operator.

While the RootBot system is designed to mimic field growing conditions by using soil or potting mix instead of liquid or agar media, it is still a more controlled and simpler environment than those found in field settings. Larger systems of this type could be constructed in a field following the RootBot's basic design. Additionally, the RootBot could easily be used with soil gathered from a specific field to examine growth in that soil while controlling for temperature and soil moisture.

## CONCLUSIONS

The RootBot system has proven to be an inexpensive and effective means of collecting root phenotyping image data in soil over the first 72 h of plant growth. Future improvements to the system could enhance its reliability and throughput. As a high‐throughput system, RootBot has the potential to image roots under large sets of environmental conditions and/or genetic variation in a relatively short period of time, potentially enabling large‐scale controlled‐environment genome‐wide association studies or other approaches that are currently limited by feasibility and the many challenges of root phenotyping.

## AUTHOR CONTRIBUTIONS

All authors contributed to the writing and revision of the manuscript. S.K.N. and M.J.O. conceived of, engineered, and developed the original RootBot system; M.R. and J.D.W. improved, tested, and refined both the hardware and software; G.S., M.M., D.K., S.K.T., and K.E.G. extensively tested the system and developed several of the data analysis pipelines. All authors approved the final version of the manuscript.

## Supporting information


**Appendix S1**. Specifications of the RootBot acrylic base.

**Appendix S2**. 3D‐printed model of a RootBot plate holder.

**Appendix S3**. Example images from RootBot.

## Data Availability

All source code, scripts, and CAD files are freely available on BitBucket (https://bitbucket.org/washjake/rootbot/). The RootBot/FarmBot OS setup, programming, and phenotype scheduling (https://doi.org/10.17504/protocols.io.x54v9d76zg3e/v1) and the image scoring protocol (https://doi.org/10.17504/protocols.io.5jyl8j16dg2w/v1) are available on protocols.io (Ruppel et al., 2023a, b).

## Appendix 1 RootBot parts list.

**Table:**

Item	Product no.	Quantity	Manufacturer/retailer
Main parts list:			
Raspberry Pi 3, Model B, ARMv8 with 1 G RAM	3055	2	Raspberry Pi Foundation
NeoPixel Ring, 24 × 5050 RGB LED with integrated drivers	1586	1	Adafruit
Adafruit Trinket, Mini Microcontroller, 5 V Logic	1501	1	Adafruit
UBEC DC/DC Step‐Down (Buck) Converter, 5 V @ 3 A output	1385	1	Adafruit
Nikon D3300 DSLR Camera with 18–55‐mm lens (black, open box)	NI1532OB	1	Nikon
Power2000 AC‐EL14 AC adapter and DC coupler kit	POACEL14	1	Power2000
SanDisk 128 GB microSDXC Memory Card Ultra Class 10 UHS‐I with SD Adapter	SAUMSD128GB	1	Western Digital
SanDisk 32 GB Ultra UHS‐I microSDHC Memory Card (Class 10)	SAUMSD32GB	1	Western Digital
SanDisk 16 GB Ultra UHS‐I microSDHC Memory Card (Class 10)	SAUMSD16GB	1	Western Digital
v1.3 Motor Kit (standard size bot)	*N/A*	1	FarmBot
0.375 Thick Black #2025 Cast Acrylic Paper‐Masked Sheet [per piece] Size: 12 × 11.02 in	*N/A*	74	Professional Plastics
0.375 Thick Black #2025 Cast Acrylic Paper‐Masked Sheet [per piece] Size: 12 × 57.06 in	*N/A*	1	Professional Plastics
0.500 Thick Black #2025 Cast Acrylic Paper‐Masked Sheet [per piece] Size: 12 × 22.41 in	*N/A*	4	Professional Plastics
0.500 Thick Black #2025 Cast Acrylic Paper‐Masked Sheet [per piece] Size: 12 × 59.06 in	*N/A*	4	Professional Plastics
0.125 Thick Black #2025 Cast Acrylic Paper‐Masked Sheet [per piece] Size: 24.41 × 59.06 in	*N/A*	1	Professional Plastics
Nunc Square BioAssay Dishes	240845	20	Thermo Scientific
PETG Filament for 3D printing	N/A	Variable	OVERTURE
Detailed Parts List (Build‐Related):			
Aluminum Flex Shaft Coupler, 5–8 mm	1176	1	Adafruit
Silicone Cover Stranded‐Core Wire, 2 m 30 AWG black	2003	1	Adafruit
Silicone Cover Stranded‐Core Wire, 2 m 30 AWG red	2001	1	Adafruit
Silicone Cover Stranded‐Core Wire, 2 m 30 AWG green	2005	1	Adafruit
Silicone Cover Stranded‐Core Wire, 2 m 30 AWG blue	2002	1	Adafruit
15 × 40 × 1000 mm Drag Chain (Cable Carrier)	30331‐03	3 ft	Inventables
SOCKiTBOX 285 Weatherproof Electrical Box, black, medium	21‐11155	1	MCM Electronics
M2.5 × 4 mm screws	91292A015	1 (25 pk)	McMaster Carr
M2.5 × 6 mm standoffs	94868A161	4	McMaster Carr
M2.5 × 16 mm standoffs	94868A171	4	McMaster Carr
M3 × 5 mm screws	92095A177	1 (100 pk)	McMaster Carr
M3 × 10 mm screws	92095A182	1 (100 pk)	McMaster Carr
M3 × 12 mm screws	92095A183	1 (100 pk)	McMaster Carr
M3 × 25 mm screws	92095A186	1 (50 pk)	McMaster Carr
M3 × 30 mm screws	92095A187	1 (50 pk)	McMaster Carr
M3 locknuts	93625A100	1 (100 pk)	McMaster Carr
M4 × 16 mm screws	91292A118	1 (100 pk)	McMaster Carr
M5 × 6 mm screws	92095A206	1 (25 pk)	McMaster Carr
M5 × 10 mm screws	92095A208	2 (100 pk)	McMaster Carr
M5 × 16 mm screws	92095A212	1 (50 pk)	McMaster Carr
M5 × 30 mm screws	92095A218	2 (25 pk)	McMaster Carr
M5 × 45 mm screws	92095A223	1 (25 pk)	McMaster Carr
M5 washers	93475A240	1 (100 pk)	McMaster Carr
M5 locknuts	93625A200	1 (100 pk)	McMaster Carr
25 mm wood screws	90252A112	1 (100 pk)	McMaster Carr
Camera mounts	8863t66	1 (5 pk)	McMaster Carr
Machine‐Mount Corrosion‐Resistant Washdown Enclosure, with gray cover	1037N92	1	McMaster Carr
Continuous Flex Cable, 300 V AC, 12 20‐gauge wires	8082k14	10	McMaster Carr
Power Cord for Tight Spaces, 8 ft long, 0.31 in OD, 16 gauge	9570T2	1	McMaster Carr
18 Gauge Easy‐ID Low‐Voltage Cable	9697t2	70	McMaster Carr
Zip ties (black)	7130K102	1 (100 pk)	McMaster Carr
3 mm hex driver	57585A55	1	McMaster Carr
2 mm hex driver	57585A53	1	McMaster Carr
8 mm box wrench	7299A23	1	McMaster Carr
5.5 mm box wrench	7299A19	1	McMaster Carr
3.5 mm Male‐to‐Male Stereo Audio Cable (8 ft)	MPC‐35‐8	1	Mediabridge
Waterproof 6 LED USB Borescope Camera, 5 m cable	9SIA5PG3C18857	1	NewEgg
Track Excrusions (20 × 40 mm V‐Slot Linear Rail)	235 ‐ LP	2	OpenBuilds
Gantry Excrusions (20 × 60 mm V‐Slot Linear Rail)	140 ‐ LP	2	OpenBuilds
M5 × 6 mm spacers	90	18	OpenBuilds
M5 × 6 mm eccentric spacers	226	15	OpenBuilds
M5 Drop‐in tee nuts	230	175	OpenBuilds
V‐wheels	55	30	OpenBuilds
Bearings	30	60	OpenBuilds
M5 shims	5	30	OpenBuilds
GT2‐2M timing belt (20 ft)	470‐By‐the‐Foot	20 ft	OpenBuilds
GT2‐2M timing pulley, 20 tooth	210	3	OpenBuilds
8 mm Metric Acme lead screw	20‐LP	1	OpenBuilds
Nut Block for 8 mm Metric Acme lead screw	740	1	OpenBuilds
12 V/29 A Meanwell Power Supply	511	1	OpenBuilds
Gantry Main Beam (20 × 60 × 620 mm V‐slot black extruded aluminum)	*N/A*	1	SMW3D
Z‐axis Excrusion (20 × 20 × 700 mm V‐slot black extruded aluminum)	*N/A*	1	SMW3D
USB to Audio Jack Sound Card Adapter	30724	1	UGREEN
