# Supplementary figures and images for: RootBot: High‐throughput root stress phenotyping robot
Source: Appl Plant Sci. 2023 Aug 28;11(6):e11541. doi: 10.1002/aps3.11541 (PMC10719875; doi:10.1002/aps3.11541)

**APPENDIX S1.** Specifications of the RootBot acrylic base.

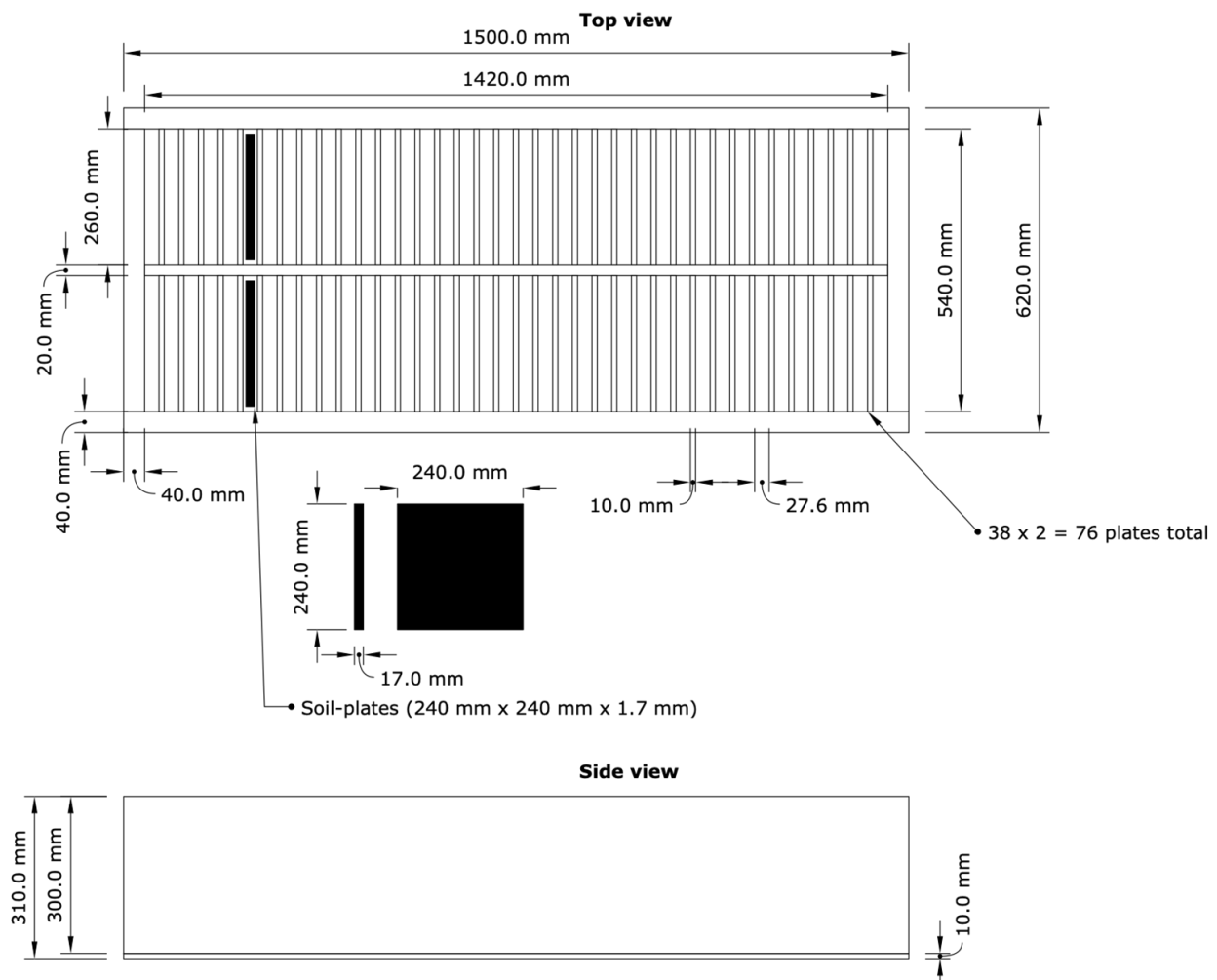

Supplement: Supplementary file 1 — Appendix S1. Specifications of the RootBot acrylic base. [file APS3-11-e11541-s002.pdf]

**APPENDIX S2.** 3D-printed model of a RootBot plate holder.

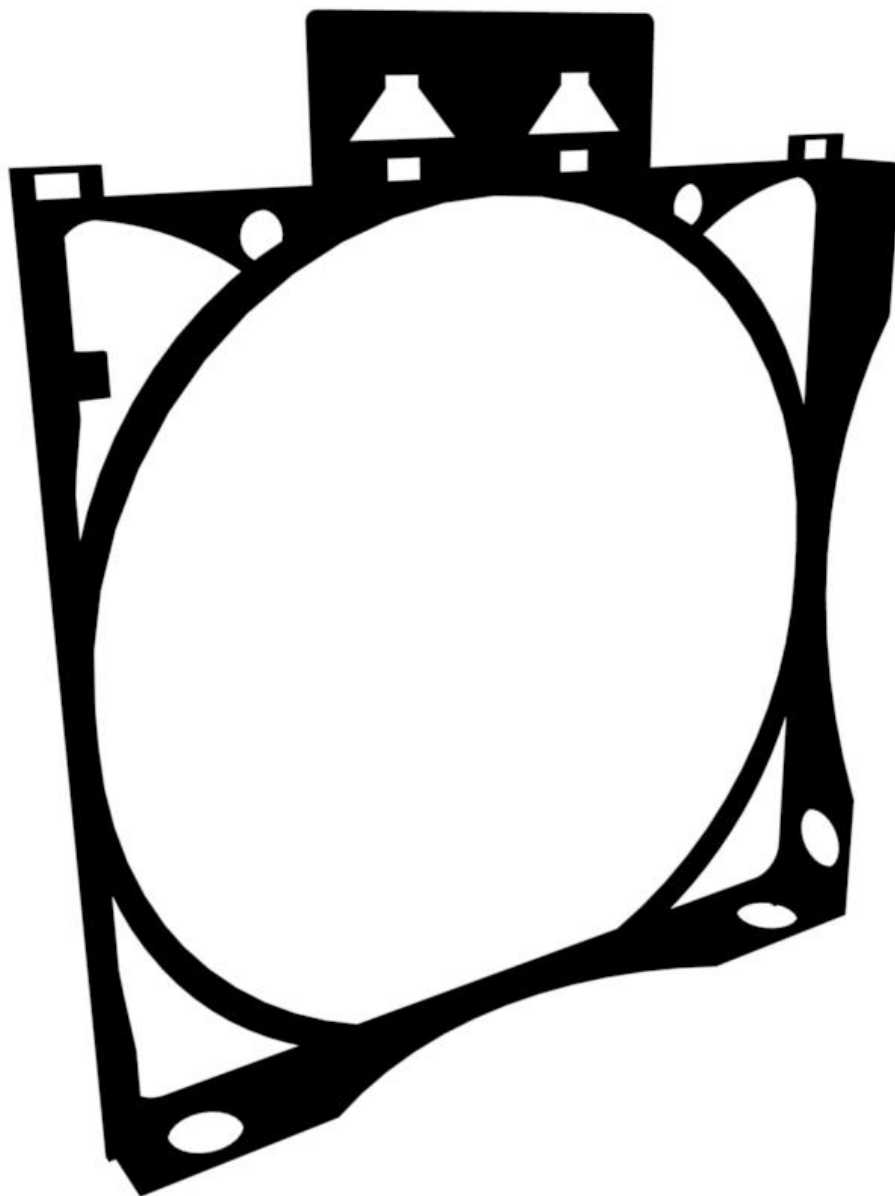

Supplement: Supplementary file 2 — Appendix S2. 3D‐printed model of a RootBot plate holder. [file APS3-11-e11541-s003.pdf]

**APPENDIX S3.** Example images from RootBot.

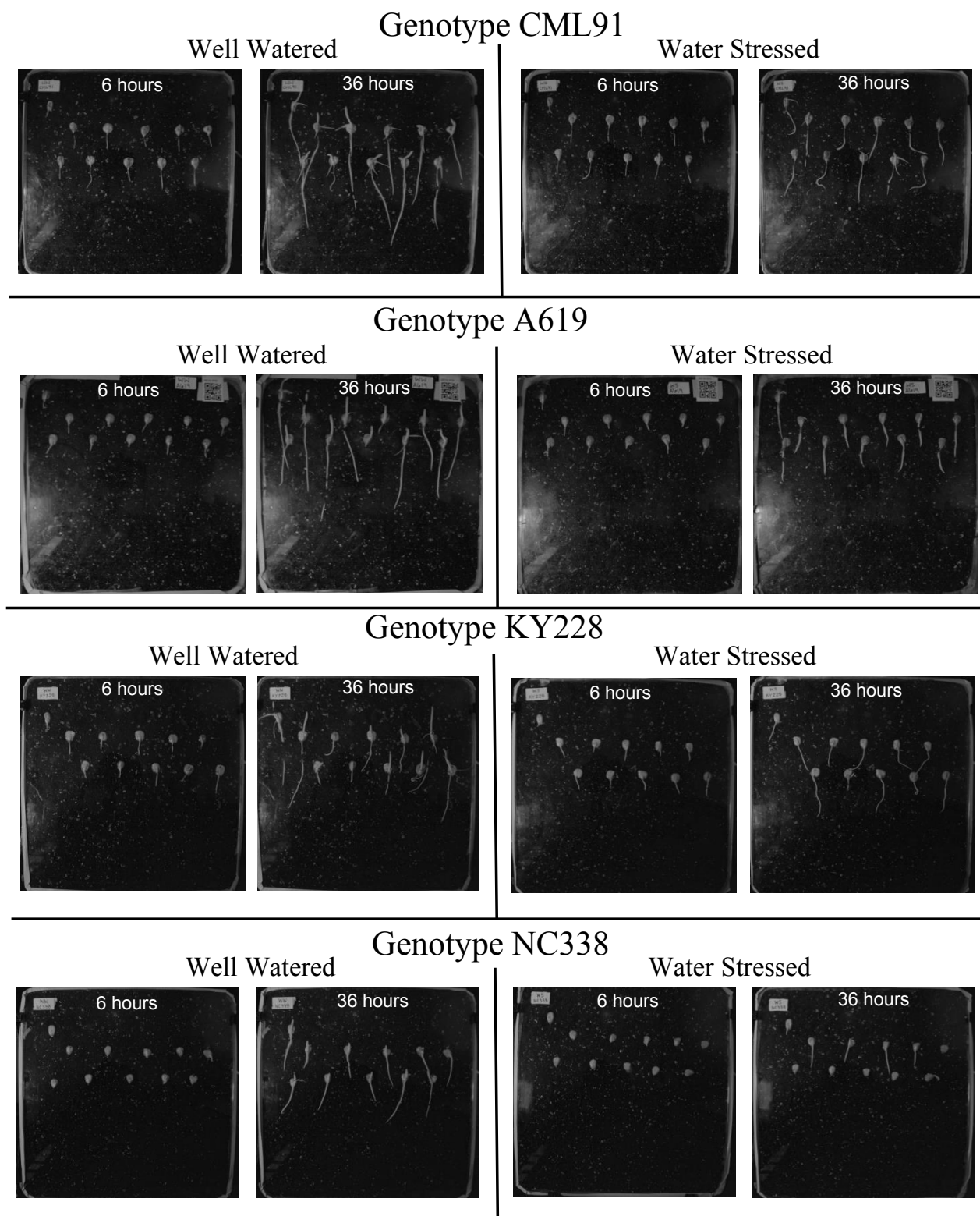

Supplement: Supplementary file 3 — Appendix S3. Example images from RootBot. [file APS3-11-e11541-s001.pdf]
